# Supplementary material for: Engagement of the G3BP2-TRIM25 Interaction by Nucleocapsid Protein Suppresses the Type I Interferon Response in SARS-CoV-2-Infected Cells
Source: Vaccines (Basel). 2022 Nov 29;10(12):2042. doi: 10.3390/vaccines10122042 (PMC9781323; doi:10.3390/vaccines10122042)
Supplement: Supplementary file 1 [file vaccines-10-02042-s001.zip › vaccines-2001470-supplementary.pdf]

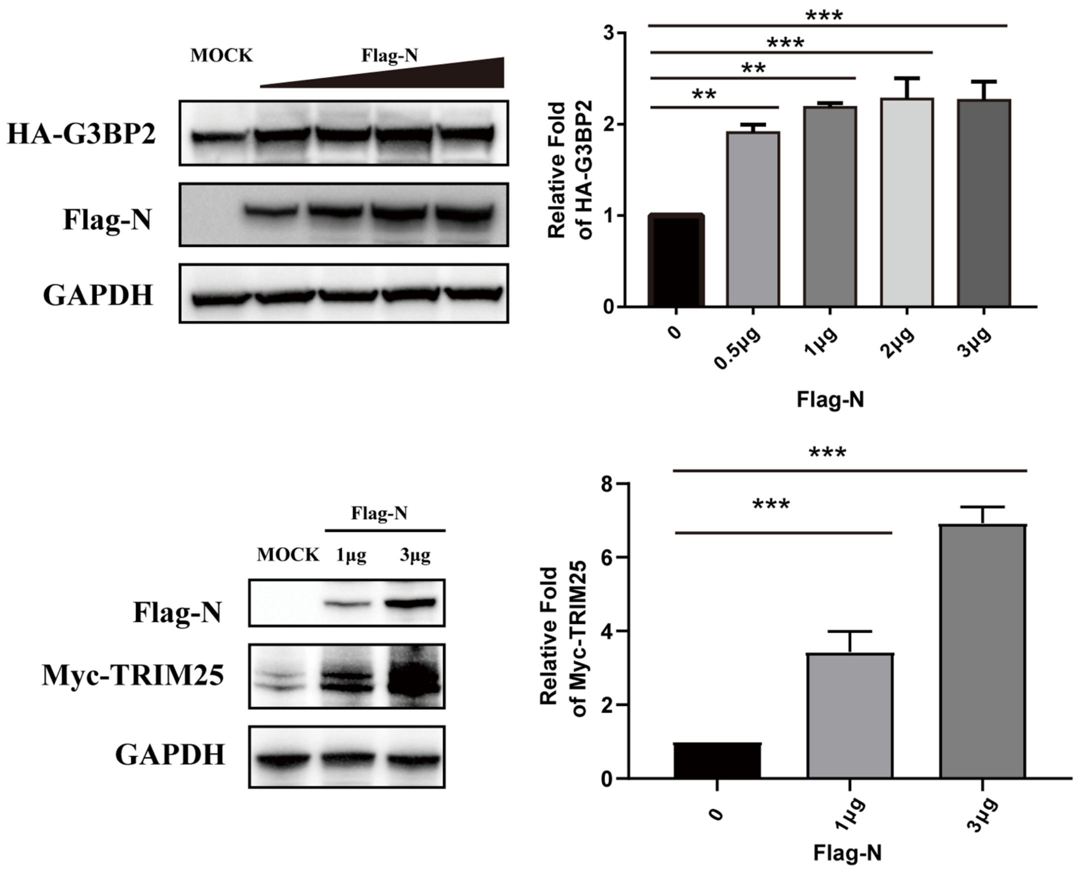

Figure S1. pcDNA3.1-G3BP2-HA or pcDNA3.1-TRIM25-Myc was cotransfected with 0.5, 1.0, 2.0, or 3.0 μg of pcDNA3.1-N-Flag. Expression of G3BP2 and TRIM25 was promoted. Grayscale results were analyzed using a Gel-Pro analyzer. Data are presented as means ± SD (paired t test, n = 3 biological replicates per group, ** 0.001 < *p* < 0.01, *** *p* < 0.001).

Table S1. MASS spectrometry results.

Number	prot_acc	prot_score	prot_mass	prot_matches	prot_cover
1	sp|P60709|ACTB_HUMAN	832	42052	26	48.8
2	sp|Q04695|K1C17_HUMAN	640	48361	22	25.5
3	sp|Q9Y606|TRUA_HUMAN	285	48010	8	17.3
4	sp|P11142|HSP7C_HUMAN	262	71082	6	11.8
5	sp|P09651|ROA1_HUMAN	259	38837	5	13.4
6	sp|Q14258|TRIM25_HUMAN	200	34686	6	10.5
7	sp|P15924|DESP_HUMAN	173	334021	6	1.9
8	sp|Q9UN86|G3BP2_HUMAN	169	54145	6	10.4
9	sp|Q7Z794|K2C1B_HUMAN	167	62149	8	5.4
10	sp|O00425|IF2B3_HUMAN	137	64008	3	7.3
11	sp|Q5T749|KPRP_HUMAN	111	67172	4	8.8
12	sp|O14602|IF1AY_HUMAN	91	16546	3	19.4
13	sp|Q8N1N4|K2C78_HUMAN	82	57629	2	4
14	sp|P84098|RL19_HUMAN	80	23565	1	8.7

15	sp|P26599|PTBP1_HUMAN	77	57357	1	4.3
16	sp|O43390|HNRPR_HUMAN	74	71184	2	3.3
17	sp|P09661|RU2A_HUMAN	73	28512	1	5.5
18	sp|Q08554|DSC1_HUMAN	71	101406	2	1.7
19	sp|Q6UWP8|SBSN_HUMAN	70	60562	1	3.1
20	sp|P14923|PLAK_HUMAN	69	82434	3	4.7
21	sp|Q96PK6|RBM14_HUMAN	56	69620	2	3.6
22	sp|P51991|ROA3_HUMAN	55	39799	1	4.2
23	sp|Q9BWM7|SFXN3_HUMAN	44	35823	1	2.8
24	sp|Q14103|HNRPD_HUMAN	44	38581	2	2.8
25	sp|Q8TAA3|PSMA8_HUMAN	43	28683	1	4.3
26	sp|P62854|RS26_HUMAN	43	13292	1	13
27	sp|P19338|NUCL_HUMAN	42	76625	1	1.1
28	sp|P46777|RL5_HUMAN	38	34569	1	3.4
29	sp|Q9BYE4|SPR2G_HUMAN	36	8779	1	17.8
30	sp|Q02880|TOP2B_HUMAN	35	184122	2	1.3
31	sp|Q7Z417|NUFP2_HUMAN	34	76132	1	1.6
32	sp|Q14028|CNGB1_HUMAN	34	140502	2	0.6
33	sp|Q99856|ARI3A_HUMAN	32	62907	1	2.2
34	sp|Q96P63|SPB12_HUMAN	32	46646	1	2.2
35	sp|Q8WWM7|ATX2L_HUMAN	32	113589	1	0.8
36	sp|P24043|LAMA2_HUMAN	31	352978	2	0.4
37	sp|Q4J6C6|PPCEL_HUMAN	31	84843	1	1.5
38	sp|P08238|HS90B_HUMAN	31	83554	1	2.1
39	sp|P48552|NRIP1_HUMAN	31	127662	1	1.3
40	sp|Q9H089|LSG1_HUMAN	30	75863	1	1.2
41	sp|O14654|IRS4_HUMAN	30	134711	1	1
42	sp|Q9NRS4|TMPS4_HUMAN	29	49127	1	2.3
43	sp|P05109|S10A8_HUMAN	29	10885	1	11.8
44	sp|Q8TCG1|CIP2A_HUMAN	27	103318	1	1.3
45	sp|Q6ZRS2|SRCAP_HUMAN	27	344996	2	0.5
46	sp|Q8NBN7|RDH13_HUMAN	27	36195	1	2.7
47	sp|Q5QNW6|H2B2F_HUMAN	27	13912	1	7.1
48	sp|Q14943|KI3S1_HUMAN	27	43131	3	2.1
49	sp|Q5GJ75|TP8L3_HUMAN	27	32867	1	2.1
50	sp|Q9UKU0|ACSL6_HUMAN	26	78728	1	1.1
51	sp|Q6Q759|SPG17_HUMAN	26	252781	1	0.4
52	sp|Q6ZR08|DYH12_HUMAN	26	359792	1	0.3
53	sp|P07384|CAN1_HUMAN	26	82465	1	2.1
54	sp|Q15758|AAAT_HUMAN	26	57018	1	2
55	sp|Q14562|DHX8_HUMAN	25	140082	1	1.3
56	sp|P49916|DNLI3_HUMAN	24	114317	1	1.7
57	sp|Q8IUS5|EPHX4_HUMAN	24	42924	1	1.9
58	sp|Q86YR7|MF2L2_HUMAN	24	128167	1	0.7
59	sp|O95096|NKX22_HUMAN	23	30171	1	2.9
60	sp|Q6AWC2|WWC2_HUMAN	23	134835	1	1.1
61	sp|Q9H3P2|NELFA_HUMAN	23	57469	1	2.1
62	sp|O00750|P3C2B_HUMAN	23	186817	1	0.7
63	sp|A6NFI3|ZN316_HUMAN	22	110422	1	0.8
64	sp|Q9BVG3|TRI62_HUMAN	22	55146	1	2.9

65	sp|Q9H5L6|THAP9_HUMAN	22	104713	1	0.9
66	sp|Q96CN9|GCC1_HUMAN	22	87985	1	1.5
67	sp|Q9H6A9|PCX3_HUMAN	21	224293	1	0.4
68	sp|Q96EM0|T3HPD_HUMAN	21	38570	1	9.3
69	sp|Q9NR20|DYRK4_HUMAN	21	60141	1	4.8
70	sp|Q07283|TRHY_HUMAN	21	254233	1	0.9
71	sp|Q9UPM8|AP4E1_HUMAN	20	128404	1	0.6
72	sp|Q9BSD7|NTPCR_HUMAN	20	20928	1	9.5
73	sp|O60447|EVI5_HUMAN	20	93460	1	1
74	sp|Q13315|ATM_HUMAN	20	355537	1	0.6
75	sp|Q75N90|FBN3_HUMAN	19	320449	1	0.4
76	sp|O43525|KCNQ3_HUMAN	18	97138	1	2.4
